# Transcriptional profiling of Petunia seedlings reveals candidate regulators of the cold stress response

**DOI:** 10.3389/fpls.2015.00118

**Published:** 2015-03-02

**Authors:** Bei Li, Luyun Ning, Junwei Zhang, Manzhu Bao, Wei Zhang

**Affiliations:** Key Laboratory of Horticultural Plant Biology, Ministry of Education, College of Horticulture and Forestry Sciences, Huazhong Agricultural UniversityWuhan, China

**Keywords:** abiotic stress, microarray, transcriptional profiling, gene expression, cold-responsive regulator, *Petunia hybrida*

## Abstract

Petunias are important ornamentals with the capacity for cold acclimation. So far, there is limited information concerning gene regulation and signaling pathways associated with the cold stress response in petunias. A custom-designed petunia microarray representing 24816 genes was used to perform transcriptome profiling in petunia seedlings subjected to cold at 2°C for 0.5 h, 2 h, 24 h, and 5 d. A total of 2071 transcripts displayed differential expression patterns under cold stress, of which 1149 were up-regulated and 922 were down-regulated. Gene ontology enrichment analysis demarcated related biological processes, suggesting a possible link between flavonoid metabolism and plant adaptation to low temperatures. Many novel stress-responsive regulators were revealed, suggesting that diverse regulatory pathways may exist in petunias in addition to the well-characterized CBF pathway. The expression changes of selected genes under cold and other abiotic stress conditions were confirmed by real-time RT-PCR. Furthermore, weighted gene co-expression network analysis divided the petunia genes on the array into 65 modules that showed high co-expression and identified stress-specific hub genes with high connectivity. Our identification of these transcriptional responses and groups of differentially expressed regulators will facilitate the functional dissection of the molecular mechanism in petunias responding to environment stresses and extend our ability to improve cold tolerance in plants.

## Introduction

The petunia (*Petunia hybrida*) is an important ornamental that is widely used in landscaping around the world. It is also an excellent model system for studying the evolutionary development of floral organs and the biosynthesis of flavonoids. Nevertheless, because the plant originated from South America, petunias are native to warm habitats. Late-spring freezes can cause substantial damage to petunias as they are less tolerant of cold conditions and lack rapid acclimation response mechanisms as compared with species tolerant of ice in their tissues, such as the spinach and Brassicas (Pennycooke et al., 2003). Thus, frozen conditions and low temperatures are significant limiting factors for its productivity, growing period and geographical location, and there is a demand for novel petunia cultivars with improved cold tolerance.

Cold stress, including chilling (<20°C) and/or freezing (<0°C) temperatures, adversely affects plant growth and development and causes a wide range of physiological and biochemical responses in plants. These responses include transient increases in hormone levels (e.g., ABA) (Chandler and Robertson, 1994), changes in the compositions of membrane lipid (Lynch and Steponkus, 1987), the accumulation of compatible osmolytes, such as soluble sugars, betaine, and praline (Koster and Lynch, 1992; Nomura et al., 1995; Doerffling et al., 1997), and increases in the level of antioxidants (Pennycooke et al., 2005). However, tolerance to freezing can be enhanced when plants are prior exposed to a period of low, but non-freezing temperatures, which is called cold acclimation (CA) (Weiser, 1970). CA activates the expression of a diverse array of plant genes (Thomashow, 2001). In the past decades, numerous cold-responsive genes have been identified and their functions have been characterized using gene transfer, resulting in increased plant stress tolerance (Sanghera et al., 2011). To date, the mechanisms of CA have been extensively investigated in the model plant *Arabidopsis thaliana* (Gilmour et al., 1998), as well as in other crucial crop species such as barley and maize (Stanca et al., 1996; Fernandes et al., 2008). The most deeply studied mechanism involves a class of ethylene response factors (ERFs), that is, CBFs (C-repeat binding factors, also called DREB1s or dehydration-responsive element-binding protein 1s), which can bind to cis-elements in the promoters of their downstream target genes to execute a highly coordinated transcriptional response to low temperature signals (Chinnusamy et al., 2007). In contrast, less information is available for petunias.

With the advancement of molecular biology technologies and “omics” tools, microarrays have been devised as a powerful strategy for the analysis of transcript abundance in plants. In recent years, transcriptional profiling of diverse plant species under defined stimuli such as hormone or abiotic stresses using microarray analysis has been particularly informative. For example, the expression patterns of genes were identified in rice and *Arabidopsis* under abscisic acid or cold, drought and high-salinity stresses (Seki et al., 2002; Rabbani et al., 2003). The transcriptomic identification of candidate genes involved in response to salt stress in cotton has also been reported (Rodriguez-Uribe et al., 2011). Additionally, transcriptome profiling of cassava treated with low temperatures highlighted the dynamic responses of tropical plants to cold stress (An et al., 2012). To date, several studies have investigated petunias using genomic tools to develop novel technologies and knowledge. For example, a cDNA library was constructed from petunias for the isolation of genes responsible for benzylbenzoate formation (Boatright et al., 2004). The capacity of a targeted transcriptomics approach to disambiguate regulation of a particular biosynthetic process was illustrated by the identification of the MYB transcription factor ODORANT1, a key regulator of floral scent biosynthesis, using a dedicated petunia microarray (Verdonk et al., 2005). Analysis based on a petunia small RNA library identified unusual tissue-specific expression patterns of conserved miRNAs and a 24-mer RNA (Tedder et al., 2009). Moreover, the petunia transcriptome has recently been employed to gain insight into potential shifts in symbiotic signaling, metabolic activity, and the defense status of plants exposed to high P levels (Breuillin et al., 2010). Nevertheless, genome-wide expression profiling data for petunias under cold stress is still deficient, thereby limiting our capacity to decipher the molecular mechanisms associated with different stress responses. To address this need for a popular ornamental, comprehensive transcriptomic data describing the response of petunias to cold stress treatment were obtained and analyzed using a custom Roche Nimblegen petunia microarray. The results revealed a set of candidate regulators. Our study provides a first glimpse into the understanding of the gene regulation pathways involved in the adaptation of this plant species to cold stress, as well as the potential applications of these data in systems biology and molecular physiology.

## Materials and methods

### Plant materials used for microarray hybridization

More than 20 inbred lines were established by successive generations of selfing. To compare the differences of cold resistance among those inbred lines, they were planted in open field in winter under natural low temperatures as well as in artificial climate chambers. One line (coded H) with the best cold tolerance was screened out.

*In vitro* seedlings of line H were planted in plastic pots at 25°C under a 14 h photoperiod with a light intensity of 2000–2500 lux provided by cool-white fluorescent lights for 1 month in the tissue culture room. Plants at 4–5 pairs of true leaf stage with a uniform growth status were transferred to a chamber for cold treatment at 2°C under weak light (cool-white fluorescent light at approximately 500–1000 lux). Whole plants were harvested after 0.5 h, 2 h, 24 h, and 5 d of treatment, then frozen in liquid nitrogen and held at −80°C prior to transportation to the Bioassay Laboratory of CapitalBio Corporation (Beijing, China) for RNA extraction. Untreated whole plants were harvested as controls (0 h). More than three plants were harvested and pooled for each time point, and the collection was repeated three times as biological replicates.

### Microarray preparation, hybridization, and data extraction

The custom-designed four-plex microarray with 72,000 features was constructed based on the EST sequences generated in Breuillin's et al. (2010) study together with all of the *P. hybrida* and *P. axillaris* sequences available from Genbank, TIGR, and the Solanaceae genomics network SGN. The compiled sequence set was ultimately composed of 24,816 non-redundant unique sequences (unigenes). Total RNA was extracted from both the control and cold-treated samples with Trizol Reagent (Invitrogen, http://www.invitrogen.com). The RNA quality was checked on a 1.2% agarose gel using an RNase-free electrophoresis system. RNA labeling and hybridization were conducted by the Bioassay Laboratory of CapitalBio Corporation (Beijing, China) following the manufacturer's instructions. Total RNA was used to generate Cy5/Cy3-labeled DNA using a cRNA amplification labeling kit (CapitalBio). Briefly, the 1st–strand cDNA was reverse transcribed with the T7 Oligo (dT) primer, then the 2nd–strand was synthesized and purified as the template for cRNA transcription with the T7 Enzyme Mix. Two micrograms of cRNA was reverse transcribed to cDNA with random primers, and the cDNA was used as the template for Cy5/Cy3-labeled DNA synthesis with Cy5/Cy3-dCTP. The Cy5/Cy3-labeled DNA in 80 μL hybridization solution (3 × SSC, 0.2% SDS, 5 × Denhart's, and 25% formamide) was hybridized to the 4 × 72K Petunia Array (Roche Nimblegen). After hybridization at 42°C overnight, the samples were washed with 2 × SSC (plus 0.2% SDS) and 0.2 × SSC for 5 min each. The GeneChips were used for scanning with the LuxScan 10 KA (CapitalBio). Three biological replicates were included in this study.

### Data collection and identification of differentially expressed genes

The probe intensity files resulting from RNA hybridization were first loaded into R. Then, background correction, quantile normalization and summarization were performed using the Robust Multi-array Average (RMA) method included in the Bioconductor Affy package (Bolstad et al., 2003; Irizarry et al., 2003; Gautier et al., 2004; Gentleman et al., 2004) based on the homemade chip description library. The correlation coefficient of paired samples was calculated to test the repeatability between samples. Then, euclidean distances were calculated, and the clustering tree was constructed using the hierarchical cluster method of “complete linkage clustering.”

Differentially expressed genes were calculated using the Bioconductor RankProd package function. RP. Rank product is a non-parametric method returning up- and down-regulated genes, their fold changes (FC), *p*-values and the percentages of false predictions (PFP). This method was shown to perform better than other methods, including significance analysis of microarrays (SAM), Fisher's Inverse χ^2^ test and t-based hierarchical modeling (Hong and Breitling, 2008). A gene was considered to be up- or down-regulated if the *p*-value of the RankProd analysis was <0.05 and the fold change of average expression was >2 (log-fold change >1 for up-regulated genes and log-fold change < −1 for down-regulated genes).

### Functional enrichment analysis

All probe set sequences were annotated by Blast2GO by searching against the nr database using Blastx with parameter *E* = 10e-3. Then, all probe sets were annotated with three principal GO categories: biological process, molecular function and cellular component. We used the terms in the biological process category for GO analysis. Finally, GO enrichment analysis was performed using a weighted method in combination with Fisher's exact test with a *P*-value of 0.05 provided by the Bioconductor topGO package (Alexa et al., 2006).

### RNA preparation and real-time RT-PCR

To validate the expression patterns of the differentially expressed genes identified from the microarray analysis, real-time RT-PCR was performed using plant materials from *in vitro* shoot cultures. *In vitro* seedlings with 4–5 pairs of true leaves were transferred into plastic jars containing 100 mL of MS solution (1 × Murashige and Skoog basal salts and 3% sucrose, pH 6.0) in a chamber at 25°C, 70% relative humidity, and a light intensity of 2000–2500 lux on a 14 h light/10 h dark cycle for 5 days. Then, the solution was replaced with fresh MS medium (pH 6.0) supplemented with 200 mM NaCl, 400 mM mannitol, 100 μM ABA, or 100 μM MeJA. For cold stress treatment, the seedlings were directly transferred into a 2°C chamber. For dehydration stress treatment, the seedlings were transferred and dehydrated onto dried filter paper in covered plastic dishes. All abiotic stress samplings were harvested from whole seedlings and frozen in liquid nitrogen after 3, 6, and 12 h and stored at −80°C for abiotic stress analysis. Plants in the same tissue culture system with no stress treatment were collected and used as controls. All of the stress treatments included three biological and temporal replicates.

The expression of cold-responsive genes was validated by real-time RT-PCR using RNA samples extracted with the EASYspin Plant RNA Mini kit according to the manufacturer's protocol (Aidlab, Beijing, China). The RNA concentration and integrity were estimated with the NanoDrop 2000 UV-Vis Spectrophotometer (Thermo scientific, USA) and denaturing agarose gel electrophoresis. Reverse transcription was performed using the Prime Script® RT reagent kit with gDNA Eraser (Perfect Real Time) (TaKaRa, Dalian, China) according to the manufacturer's protocol. Gene-specific primers (Table S1) were designed with ABI primer express 3.0 software (Applied Biosystems, USA). The product size from each amplicon ranged from 105 to 215 base pairs. The real-time RT-PCR was performed with SYBR® Premix Ex Taq™ (Perfect Real Time) (TaKaRa, Dalian, China) as described in the users' instructions for the ABI 7500 Fast Real-Time PCR System (Applied Biosystems, USA). The PCR reaction mixture contained 1 μl cDNA template, 5 μl SYBR®Premix Ex Taq™ (2x), 0.2 μl forward and reverse primer (10 μmol/μl each), 0.2 μl ROX Reference Dye II (50x), and water up to 10 μl. The amplification conditions were as follows: an initial incubation at 95°C for 30 s, followed by 40 cycles of 95°C for 3 s, 60°C for 30 s, and 72°C for 20 s. A melting curve (60°–95°C with a heating rate of 0.05°C·s^−1^ and a continuous fluorescence measurement) was performed for each qRT-PCR reaction to determine PCR performances. The glyceraldehyde-3-phosphatedehydrogenase gene (*GAPDH*) was used as the internal control. All of the samples were measured in triplicate, and the experiments were performed on three biological replicates. The comparative Ct method was adopted to calculate the relative gene expression level across the samples. The relative expression level of each gene in one sample (ΔCt) was calculated as follows: Ct target gene – Ct GAPDH. The relative expression of each gene in two diverse samples (ΔΔCt) was calculated as follows: ΔCt (sample 1) – ΔCt (sample 2).

### Co-expression network analysis

To identify co-expression of the petunia genes on the array, log-transformed gene expression values for each condition were used in a meta-analysis to perform Weighted Gene Co-expression Network Analysis (WGCNA) (Langfelder and Horvath, 2008). Briefly, WGCNA can be used for finding clusters (modules) of highly correlated genes, for summarizing such clusters using the module eigengene or an intramodular hub gene, for calculating module membership measures, and for relating modules to one another and to external sample traits (using eigengene network methodology). The blockwiseModules function of the WGCNA package in R was used to generate the modules with a power of six. The correlation coefficient relationship of the module eigengene was calculated, then euclidean distances were calculated and the clustering tree was constructed using the hierarchical cluster method of “complete linkage clustering” included with the R package.

## Results

**Figure 1:**
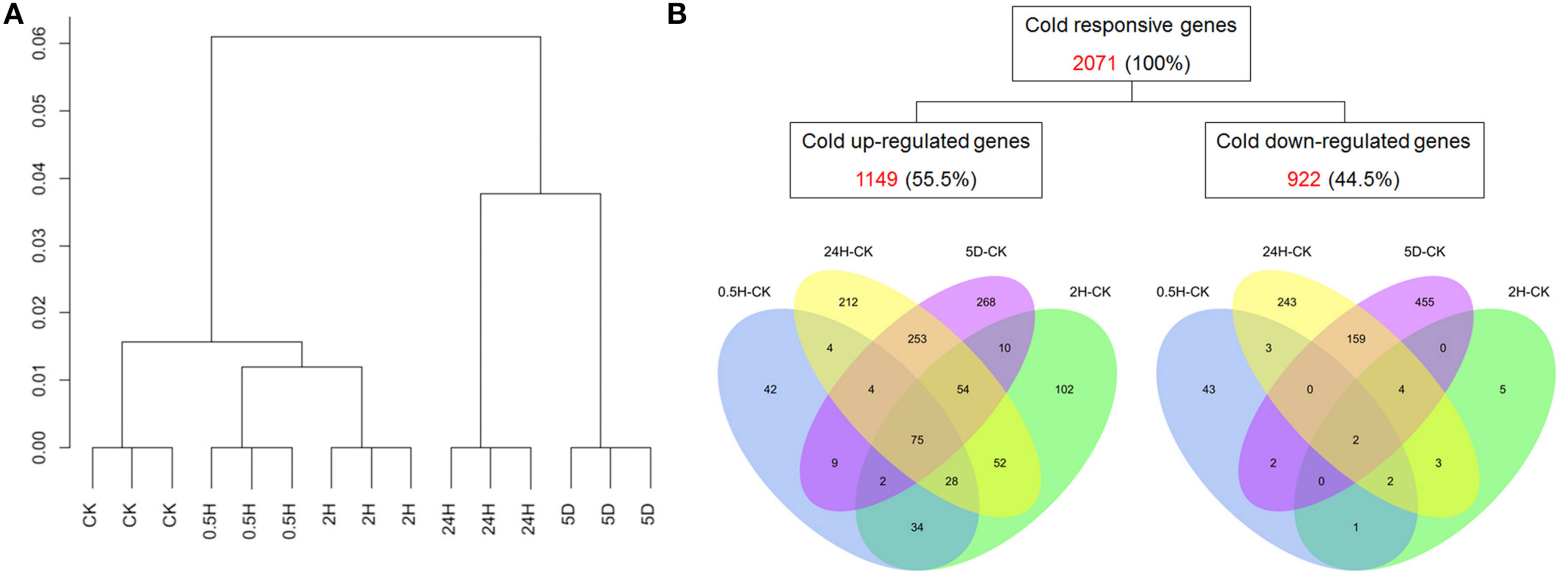
**Expression profiling of cold-regulated genes in petunia seedlings. (A)** Hierarchical cluster analysis. **(B)** Venn diagrams showing cold-regulated genes across four comparisons (0.5 h/0 h, 2 h/0 h, 24 h/0 h, and 5 d/0 h). The red numbers are the total numbers of differentially expressed genes (DEGs); the percentages in parentheses were calculated as the ratio of regulated genes to the total number of cold-regulated genes (2071).

To explore the transcriptomic changes in petunias in response to cold stress, a custom four-plex microarray with 72,000 features generated by Roche NimbleGen was employed in the current study. The detailed protocol for designing the petunia microarray has been described by Breuillin et al. (2010). Our microarray data have been deposited to Gene Expression Omnibus (http://www.ncbi.nlm.nih.gov/geo/query/acc.cgi?acc=GSE64963) and the accession number is GSE64963. The results of hierarchical clustering indicated the clear separation of the samples from various time points (Figure 1A), which suggested that the whole experiment from sample collection to data extraction was reliable and reproducible.

### Transcriptomic responses of petunia seedlings to cold treatment

Two filtering criteria were used in our data analysis to define differentially expressed genes (DEGs): a two-fold or greater change in transcript levels between any two time points and a *P*-value <0.05. To analyze the differences and similarities among the cold-responsive transcriptomes, a hierarchical clustering approach was utilized to depict the transcripts of all of the differentially expressed probes of the three replicates at 0 h, 0.5 h, 2 h, 24 h, and 5 d. These results demonstrated distinct differences in gene expression profiles between the treatments of 5 d and 0 h or 24 h and 0 h that were in contrast to the relatively highly similar expression profiles between 2 h and 0 h or 0.5 h and 0 h (Figure 1A). Among the DEGs, 198, 357, 682, and 675 genes were considered to be up-regulated and 53, 17, 416, and 622 genes were considered to be down-regulated based on the analysis of 0.5 h/0 h, 2 h/0 h, 24 h/0 h, and 5 d/0 h, respectively (Table S2). Notably, compared with only 251 and 374 genes that showed differential expression at 0.5 h/0 h and 2 h/0 h, 1098, and 1297 genes were found to be differentially expressed at 24 h/0 h and 5 d/0 h, suggesting that prolonged stress treatment triggered the expression of more stress-related genes. To identify both unique and common genes showing differential expression patterns at all time points compared with 0 h, the numbers were calculated and presented using a Venn diagram (Figure 1B). The results showed that 75 DEGs were commonly induced in all of the comparisons for each time point compared with 0 h, which demonstrated a strong linkage among the four stressed comparison points and a progressive biological process. In contrast, 42, 102, 212, and 268 DEGs exhibiting up-regulated expression were unique at 0.5 h/0 h, 2 h/0 h, 24 h/0 h, and 5 d/0 h; thus, these genes may play special roles at those time points. Only 2 suppressed DEGs were found to be common to all of the comparisons for each time point compared with 0 h, whereas approximately 81% (43/53), 29% (5/17), 58% (243/416), and 73% (455/622) of DEGs exhibiting down-regulated expression were unique at 0.5 h/0 h, 2 h/0 h, 24 h/0 h, and 5 d/0 h, respectively.

In total, 2071 DEGs, including 1149 cold-inducible and 922 cold-repressed genes, were identified using our array. These genes represent approximately 8.3% (2071/24,816) of the total expressed petunia transcripts on the array. This result indicated that *Petunia hybrida* is a species with the capacity for cold acclimation (Yelenosky and Guy, 1989) that possess a similarly large amount of cold-responsive genes similar to that reported for other plants (e.g., *Arabidopsis*). Indeed, the petunia arrays employed here included probes for only a portion of the total genes. Thus, if the probes on the array covered the whole genome, many more genes responsive to cold would have been found. Because differences in the ability of diverse plant species to tolerate cold might not be totally due to the amount of genes responsive to cold stress (Carvallo et al., 2011), it has been suggested that plants require to duly and coordinately mobilize the biological functions and regulatory networks of stress-responsive genes.

### Comparison of significant gene ontology terms of DEG sets at all time points

To gain insight into the putative functions of the 2071 cold-responsive genes identified by the microarray analysis, gene ontology annotation for all of the differentially expressed genes was performed using Blast2gGO against the local nr database. Accordingly, 1388 annotations were obtained based on the gene enrichment analysis that was performed for each comparison group between all time points and 0 h. All three principal GO categories (“Biological Process,” “Molecular Function,” and “Cellular Component”) were collected for each gene, and the biological process was selected for functional enrichment analysis.

We found 198 unique GO terms enriched by DEGs when analyzed gene sets that were up- or down-regulated separately at each time point (Table S3). At 0.5 h/0 h, the most significant GO terms found in the up-regulated gene set were regulation of RNA biosynthetic process (GO:2001141), regulation of RNA metabolic process (GO:0051252) and regulation of transcription, DNA-templated (GO:0006355). The most significant GO terms in the down-regulated sets were transition metal ion transport, and especially zinc ion transmembrane transport (e.g., GO:0000041 and GO:0071577). At 2 h/0 h, only the up-regulated gene sets could be enriched for GO analysis. These DEGs were mainly involved in positive regulation of nucleobase-containing compound metabolic process (GO:0045935), positive regulation of gene expression (GO:0010628), positive regulation of RNA metabolic process (GO:0051254) and positive regulation of transcription, DNA-templated (GO:0045893). The functional enrichment analysis for the down-regulated gene sets was not available, partially due to the lack of annotation for a number of petunia genes. At 24 h/0 h, the terms, “flavone metabolic process” (GO:0051552), “pigment biosynthetic process” (GO:0046148), “flavonol metabolic process” (GO:0051554), “flavone biosynthetic process” (GO:0051553), and “flavonol biosynthetic process” (GO:0051555) were significantly enriched in the up-regulated gene sets (shown in Table 1), whereas responses to various abiotic stresses such as oxygen-containing compound, alcohol and lipid (GO:1901700, GO:0097305 and GO:0033993) and “jasmonic acid biosynthetic process” (GO:0009695) were significantly enriched in the down-regulated gene sets. The top significant GO terms found in the up-regulated gene sets at 5 d/0 h were identical to those at 24 h/0 h (Table S3), whereas the terms significantly enriched in the down-regulated gene sets at 5 d/0 h were mainly represented by response to oxygen-containing compound (GO:1901700).

**Table 1:** **Gene ontology (GO) terms enriched by cold induced genes at 24 h/0 h**.

**GO.ID**	**Term**	**Level**	**Annotated**	**Significant**	**Expected**	**Classic**	**Probe ID**
GO:0051552	Flavone metabolic process	5	2	2	0.04	0.00042	cn2956, cn2958
GO:0051553	Flavone biosynthetic process	6	2	2	0.04	0.00042	cn2956, cn2958
GO:0051554	Flavonol metabolic process	6	2	2	0.04	0.00042	cn2956, cn2958
GO:0051555	Flavonol biosynthetic process	7	2	2	0.04	0.00042	cn2956, cn2958
GO:0046148	Pigment biosynthetic process	5	10	3	0.21	0.00085	cn173, cn2956, cn2958
GO:0042440	Pigment metabolic process	4	11	3	0.23	0.00116	cn173, cn2956, cn2958
GO:0009813	Flavonoid biosynthetic process	5	13	3	0.27	0.00195	cn2956, cn2958, cn3395
GO:0009812	Flavonoid metabolic process	4	14	3	0.29	0.00245	cn2956, cn2958, cn3395
GO:0048518	Positive regulation of biological process	4	14	3	0.29	0.00245	cn173, cn4309, cn8214
GO:0010628	Positive regulation of gene expression	7	5	2	0.1	0.004	cn4309, cn8214
GO:0045893	Positive regulation of transcription, DNA-templated	11	5	2	0.1	0.004	cn4309, cn8214
GO:0045935	Positive regulation of nucleobase-containing compound metabolic process	7	5	2	0.1	0.004	cn4309, cn8214
GO:0051173	Positive regulation of nitrogen compound metabolic process	6	5	2	0.1	0.004	cn4309, cn8214
GO:0051254	Positive regulation of RNA metabolic process	9	5	2	0.1	0.004	cn4309, cn8214
GO:1902680	Positive regulation of RNA biosynthetic process	10	5	2	0.1	0.004	cn4309, cn8214
GO:0009891	Positive regulation of biosynthetic process	6	6	2	0.13	0.00592	cn4309, cn8214
GO:0010557	Positive regulation of macromolecule biosynthetic process	7	6	2	0.13	0.00592	cn4309, cn8214
GO:0010604	Positive regulation of macromolecule metabolic process	6	6	2	0.13	0.00592	cn4309, cn8214
GO:0031328	Positive regulation of cellular biosynthetic process	7	6	2	0.13	0.00592	cn4309, cn8214
GO:0009893	Positive regulation of metabolic process	5	7	2	0.15	0.00819	cn4309, cn8214
GO:0031325	Positive regulation of cellular metabolic process	6	7	2	0.15	0.00819	cn4309, cn8214
GO:0071478	Cellular response to radiation	5	8	2	0.17	0.01078	cn172, cn173
GO:0071482	Cellular response to light stimulus	6	8	2	0.17	0.01078	cn172, cn173
GO:0048522	Positive regulation of cellular process	5	10	2	0.21	0.0169	cn4309, cn8214
GO:0071214	Cellular response to abiotic stimulus	4	10	2	0.21	0.0169	cn172, cn173
GO:0009737	Response to abscisic acid	6	11	2	0.23	0.0204	cn4309, cn8214
GO:0009960	Endosperm development	10	1	1	0.02	0.02093	cn6513
GO:0010029	Regulation of seed germination	8	1	1	0.02	0.02093	cn173
GO:0010030	Positive regulation of seed germination	9	1	1	0.02	0.02093	cn173
GO:0010114	Response to red light	7	1	1	0.02	0.02093	cn173
GO:0010117	Photoprotection	6	1	1	0.02	0.02093	cn173
GO:0010380	Regulation of chlorophyll biosynthetic process	9	1	1	0.02	0.02093	cn173
GO:0034605	Cellular response to heat	6	1	1	0.02	0.02093	cn173
GO:0043248	Proteasome assembly	8	1	1	0.02	0.02093	cn6513
GO:0051193	Regulation of cofactor metabolic process	6	1	1	0.02	0.02093	cn173
GO:0051788	Response to misfolded protein	6	1	1	0.02	0.02093	cn6513
GO:0070141	Response to UV-A	7	1	1	0.02	0.02093	cn173
GO:0071486	Cellular response to high light intensity	8	1	1	0.02	0.02093	cn173
GO:0071490	Cellular response to far red light	8	1	1	0.02	0.02093	cn173
GO:0071491	Cellular response to red light	8	1	1	0.02	0.02093	cn173
GO:0071492	Cellular response to UV-A	8	1	1	0.02	0.02093	cn173
GO:0090056	Regulation of chlorophyll metabolic process	8	1	1	0.02	0.02093	cn173
GO:1900140	Regulation of seedling development	7	1	1	0.02	0.02093	cn173
GO:1901401	Regulation of tetrapyrrole metabolic process	6	1	1	0.02	0.02093	cn173
GO:1901463	Regulation of tetrapyrrole biosynthetic process	7	1	1	0.02	0.02093	cn173
GO:1901617	Organic hydroxy compound biosynthetic process	5	13	2	0.27	0.02822	cn2956, cn2958
GO:0009639	Response to red or far red light	6	14	2	0.29	0.03251	cn172, cn173
GO:0006511	Ubiquitin-dependent protein catabolic process	11	15	2	0.31	0.03705	cn2609, cn6513
GO:0019941	Modification-dependent protein catabolic process	10	15	2	0.31	0.03705	cn2609, cn6513
GO:0043632	Modification-dependent macromolecule catabolic process	7	15	2	0.31	0.03705	cn2609, cn6513
GO:0006508	Proteolysis	8	37	3	0.77	0.03848	cn2609, cn6513, GO_drpoolB-CL4454Contig1
GO:0016485	Protein processing	7	37	3	0.77	0.03848	cn2609, cn6513, GO_drpoolB-CL4454Contig1
GO:0051604	Protein maturation	6	37	3	0.77	0.03848	cn2609, cn6513, GO_drpoolB-CL4454Contig1
GO:0001558	Regulation of cell growth	5	2	1	0.04	0.04143	cn2956
GO:0022604	Regulation of cell morphogenesis	7	2	1	0.04	0.04143	cn173
GO:0034644	Cellular response to UV	7	2	1	0.04	0.04143	cn173
GO:0035966	Response to topologically incorrect protein	5	2	1	0.04	0.04143	cn2956
GO:0040008	Regulation of growth	4	2	1	0.04	0.04143	cn2956
GO:0042023	DNA endoreduplication	7	2	1	0.04	0.04143	cn173
GO:0044786	Cell cycle DNA replication	6	2	1	0.04	0.04143	cn4309
GO:0048638	Regulation of developmental growth	5	2	1	0.04	0.04143	cn4309
GO:0051510	Regulation of unidimensional cell growth	8	2	1	0.04	0.04143	cn4309
GO:0071484	Cellular response to light intensity	7	2	1	0.04	0.04143	cn4309
GO:0097305	Response to alcohol	5	16	2	0.33	0.04182	cn4309, cn8214
GO:0018130	Heterocycle biosynthetic process	5	130	6	2.72	0.04235	cn10124, cn173, cn2463, cn4309, cn559, cn8214
GO:0044271	Cellular nitrogen compound biosynthetic process	5	130	6	2.72	0.04235	cn10124, cn173, cn2463, cn4309, cn559, cn8214
GO:0051171	Regulation of nitrogen compound metabolic process	5	67	4	1.4	0.04514	cn10124, cn173, cn4309, cn8214

### Transcription factor responses to cold stress

We surveyed the putative transcription factors (TFs) that were differentially expressed in petunias at one or more time points under cold treatment based on the annotations (Table S2); a total of 61 DGEs were identified as TFs involved in the cold stress response. The number of up-regulated TFs (48) was prominently greater than the number of down-regulated TFs (13), suggesting that transcriptional activation but not repression was involved. There are more than 2657 predicted TFs in the *Arabidopsis* genome belonging to 81 gene families that are included in the Plant Transcription Factor Database (PlnTFDB, http://plntfdb.bio.uni-potsdam.de/v3.0/index.php?sp_i-d=ATH) (Perez-Rodriguez et al., 2010). In accordance with this classification, the 61 cold-responsive transcription factors fell into 12 families (Table 2). The six major TF families included AP2-EREBP (APE-TALA2/ET-Responsive Element Binding Protein), zinc finger, MADS-box (MCMI, AG, DEFA, and SRF), MYB (Myeloblastosis)/MYB-related, NAC (NAM, ATAF1/2, CUC2) and GRAS (GAI, RGA, SCR), containing 15, 11, 9, 9, 5, and 5 genes of the 61 total cold-responsive TFs, respectively.

**Table 2:** **Responsive transcription factors during the process of low temperature treatment in petunia seedlings**.

**Family**	**Probe ID**	**0.5 h/0 h**	**2 h/0 h**	**24 h/0 h**	**5 d/0 h**	**Best hit in the protein database (blastx)**		
						**Accession**	***E*-value**	**Annotation**
AP2-EREBP	cn2811	–	–	2.38	–	NP_200015	1.00E-17	ERF025 (ethylene-responsive transcription factor) [*Arabidopsis thaliana*]
AP2-EREBP	cn4173	–	–	−1.87	−3.45	NP_197901	8.00E-33	ERF003 (ethylene-responsive transcription factor) [*Arabidopsis thaliana*]
AP2-EREBP	cn4335	–	1.34	–	–	AAX20035	7.00E-66	Ethylene responsive element binding protein C2 [*Capsicum annuum*]
AP2-EREBP	cn6160	–	–	3.32	–	EEF33061	1.00E-21	Ethylene-responsive transcription factor, putative [*Ricinus communis*]
AP2-EREBP	cn9604	1.21	2.56	1.88	–	Q9LW49	1.00E-24	ERF3(ethylene-responsive element binding factor)[*Nicotiana sylvestris*]
AP2-EREBP	DY395831_1	–	2.58	5.39	–	BAF48804	8.00E-16	Wound-responsive AP2 like factor 2 [*Nicotiana tabacum*]
AP2-EREBP	GO_dr001P0011E21_F_ab1	–	–	−2.16	−3.39	AAU81956	2.00E-32	ERF5 [*Nicotiana tabacum*]
AP2-EREBP	GO_dr001P0015C16_F_ab1	–	1.32	2.00	1.65	Q9LW49	2.00E-40	ERF3 (ethylene-responsive element binding factor) [*Nicotiana sylvestris*]
AP2-EREBP	GO_dr001P0017B08_F_ab1	−3.12	–	–	–	ABK96798	7.00E-24	ERF3 (ethylene response factor 3) [*Solanum tuberosum*]
AP2-EREBP	GO_dr004P0008C18_F_ab1	–	–	2.42	–	EEF46968	1.00E-27	DNA binding protein, putative [*Ricinus communis*]
AP2-EREBP	GO_dr004P0029M08_F_ab1	3.58	6.66	5.53	4.35	AAQ88400	6.00E-65	CBF1B [*Capsicum annuum*]
AP2-EREBP	GO_drpoolB-CL6306Contig1	–	–	2.18	2.73	AAU81956	8.00E-33	ERF5 [*Nicotiana tabacum*]
AP2-EREBP	GO_drpoolB-CL6454Contig1	–	1.33	–	–	AAO13360	1.00E-36	DREB3(dehydration-responsive element binding protein 3)[*Lycopersicon esculentum*]
AP2-EREBP	GO_drpoolB-CL7405Contig1	–	1.33	–	–	AAO13360	3.00E-17	Dehydration-responsive element binding protein 3 [*Lycopersicon esculentum*]
AP2-EREBP	IP_PHBS008P03u	3.18	6.23	–	–	ABU84809	4.00E-15	Putative dehydration-responsive element binding protein [*Broussonetia papyrifera*]
AUX/IAA	cn8979	–	–	4.41	–	EEF44693	3.00E-49	Auxin-responsive protein IAA1, putative [*Ricinus communis*]
GRAS	cn10124	–	–	2.09	–	ABD72959	1.00E-118	GRAS2 [*Solanum lycopersicum*]
GRAS	GO_drpoolB-CL9151Contig1	–	–	−1.86	–	ABJ51763	8.00E-51	NSP1-like (nodulation signaling pathway 1-like protein)[*Nicotiana benthamiana*]
GRAS	GO_drs12P0009O19_F_ab1	–	–	2.12	–	ABD72958	2.00E-90	GRAS1 [*Solanum lycopersicum*]
GRAS	GO_drs12P0009O19_R_ab1	–	–	2.58	–	ABD72958	1.00E-64	GRAS1 [*Solanum lycopersicum*]
GRAS	GO_drs13P0003F13_R_ab1	–	1.38	3.03	–	ACB30358	9.00E-34	Putative scarecrow protein [*Capsicum annuum*]
HLH	cn3063	–	–	−1.92	−2.85	ABX82930	5.00E-11	transcription Factor style2.1 [*Solanum lycopersicum*]
HLH	EB174526_1	–	–	−1.94	–	EEF41534	6.00E-42	DNA binding protein, putative [*Ricinus communis*]
HSF	DY396073_1	–	–	3.77	4.49	AAM43804	9.00E-33	HSFA9(heat stress transcription factor) [*Helianthus annuus*]
MADS-box	cn2934	1.64	–	–	–	AAA68001	1.00E-123	Agamous protein (*Petunia inflata*)
MADS-box	cn300	1.85	–	–	–	Q40885	1.00E-116	pMADS3 [Petunia x hybrida]
MADS-box	cn435	3.34	–	–	2.85	Q07474	1.00E-116	pMADS2 [Petunia x hybrida]
MADS-box	cn704	1.82	–	–	–	Q07472	1.00E-113	pMADS1 [Petunia x hybrida]
MADS-box	cn716	3.48	–	–	3.34	Q03489	1.00E-126	FBP2 (Floral-binding protein 2) [Petunia x hybrida]
MADS-box	cn748	1.85	–	–	–	AAK21254	1.00E-125	FBP23 [Petunia x hybrida]
MADS-box	cn80	4.48	–	–	3.43	AAS46018	1.00E-114	GLO1 [Petunia x hybrida]
MADS-box	GI_NP1240016	–	−2.53	−2.63	–	AAK21253	1.00E-107	FBP22 [Petunia x hybrida]
MADS-box	GI_NP1240120	–	1.46		2.27	CAA57445	1.00E-124	FBP11 [Petunia x hybrida]
MYB	cn2063	–	–	2.59	–	BAF96932	2.00E-58	R2R3-MYB transcriptional factor [*Gentiana triflora*]
MYB	cn6894	–	–	−2.69	–	EEF48780	3.00E-70	R2R3-myb transcription factor, putative [*Ricinus communis*]
MYB	cn8223	–	–	4.74	7.10	ABY40371	4.00E-53	MYB transcription factor [*Solanum tuberosum*]
MYB	GO_dr004P0032H09_F_ab1	–	1.38	–	–	EEF47869	1.00E-42	R2R3-myb transcription factor, putative [*Ricinus communis*]
MYB	GO_drpoolB-CL7385Contig1	–	–	−2.18	–	AAG08962	9.00E-98	Tuber-specific and sucrose-responsive element binding factor [*Solanum tuberosum*]
MYB-related	cn5031	–	–	1.97	–	CAB65169	4.00E-36	I-box binding factor [*Solanum lycopersicum*]
MYB-related	GO_dr001P0009H07_F_ab1	–	2.83	4.05	4.54	EEF32969	3.00E-84	DNA binding protein, putative [*Ricinus communis*]
MYB-related	GO_dr004P0014N06_F_ab1	–	–	−2.29	−3.02	BAE93149	3.00E-66	methyl jasmonate induced MYB-related transcription factor [*Nicotiana tabacum*]
MYB-related	GO_dr004P0017J05_F_ab1	–	–	−1.72	–	BAA88222	1.00E-51	LBM2 [*Nicotiana tabacum*]
NAC	cn3313	–	–	2.55	–	AAM50520	0	nam-like protein 17 [Petunia x hybrida]
NAC	cn3314	–	–	−1.72	–	AAM50518	1.00E-143	nam-like protein 15 [Petunia x hybrida]
NAC	GI_NP1239993	–	–	1.98	–	AAM34766	1.00E-177	nam-like protein 3 [Petunia x hybrida]
NAC	GO_drpoolB-CL3154Contig1	–	2.35	5.47	5.90	EEF28468	6.00E-20	NAC domain-containing protein, putative [*Ricinus communis*]
NAC	SG_SGN-U211561	–	–	2.39	–	EEF32064	9.00E-14	NAC domain-containing protein, putative [*Ricinus communis*]
SBP-box	cn10022	–	–	2.32	–	EEF50305	7.00E-48	LIGULELESS1 protein, putative [*Ricinus communis*]
SBP-box	cn8031	–	–	2.57	–	EEF50305	9.00E-11	LIGULELESS1 protein, putative [*Ricinus communis*]
TCP	EB174538_1	–	−1.88	–	–	ABW08044	4.00E-26	Eukaryotic transcription factor [Conandron ramondioides]
Zinc finger protein (C2H2-type)	cn3341	–	–	4.07	3.86	BAA21925	1.00E-91	ZPT2-8 [Petunia x hybrida]
Zinc finger protein (C2H2-type)	cn3342	–	–	3.76	3.64	BAA21924	7.00E-68	ZPT2-7 [Petunia x hybrida]
Zinc finger protein (C2H2-type)	cn3346	1.84	4.31	4.44	3.31	BAA21921	3.00E-77	ZPT2-12 [Petunia x hybrida]
Zinc finger protein (C2H2-type)	cn762	–	–	2.09	–	BAA05077	3.00E-85	Zinc-finger DNA binding protein [Petunia x hybrida]
Zinc finger protein (C2H2-type)	GI_NP1240035	1.62	3.03	6.31	4.41	BAA21926	1.00E-86	ZPT2-9 [Petunia x hybrida]
Zinc finger protein (C2H2-type)	GI_NP1240041	–	1.29	–	–	BAA21920	1.00E-131	ZPT2-11 [Petunia x hybrida]
Zinc finger protein (C2H2-type)	GI_NP1240214	–	–	2.81	–	BAA05078	1.00E-115	Zinc-finger DNA binding protein [Petunia x hybrida]
Zinc finger protein (C2H2-type)	GI_NP1240263	–	1.28	–	–	CAA43111	1.00E-143	DNA-binding protein [Petunia x hybrida]
Zinc finger protein (C2H2-type)	**GO_drpoolBCL3762Contig1**	2.53	3.68	2.73	1.02	BAA05079	4.00E-35	zinc-finger protein [Petunia x hybrida]
Zinc finger protein(C3H-type)	GO_drs31P0004P18_R_ab1	–	2.19	2.63	1.54	EEF34053	9.00E-34	Transcription factor, putative [*Ricinus communis*]
Zinc finger protein (B-box-type)	GO_drs21P0009P06_F_ab1	–	–	1.91	3.70	EEF37806	2.00E-38	Transcription factor, putative [*Ricinus communis*]

*All data are shown log2 Ratio, and positive and negative values of Log2 Ratio are either up- or down-regulated genes in the four pair-comparisons. No significant fold changes are indicated as “–.” One probe ID showing in bold font indicates the gene PhZFP1*.

Members of the AP2-EREBP family belong to different subfamilies of the ERF/AP2 TF family. For example, *AtERF025*, *AtERF003*, *CaEREBP-C2*, and *NsERF3* encode members of the A-4, B-6, B-3, and B-1 subfamilies of the ERF/AP2 TF family. Importantly, we observed that more than half of the members of this family were diversely induced at the early stage (0.5 h or 2 h) during cold treatment, suggesting a relatively fast reaction in triggering transcriptional cascades in the cold response of petunias. We arbitrarily selected two AP2/EREBP TFs (cn9604 and GO_dr001P0015C16_F_ab1) for validation by qRT-PCR. As shown in Table 3, both microarray and qRT-PCR analyses were in agreement concerning the substantial inducibility of the transcripts of these two members following cold treatment. Moreover, the highly induced expression of one *CBF* homologous gene (GO_dr004P0029M08_F_ab1), named *PhCBF1* hereafter, was also verified by real-time RT-PCR (Table 3), implying that the timely response of the DREB/CBF pathway to cold might be similar at least in part between petunias and other plants.

**Table 3:** **Validation of selected array-based gene expression by real-time RT-PCR analysis**.

**Probe ID**	**Description**	**Microarray**				**qRT–PCR**			
		**0.5 h/0 h**	**2 h/0 h**	**24 h/0 h**	**5 d/0 h**	**0.5 h/0 h**	**2 h/0 h**	**24 h/0 h**	**5 d/0 h**
GO_dr004P0029M08_F_ab1	CBF1	3.58	6.66	5.53	4.35	5.41	6.07	6.83	3.73
cn9604	AP2-EREBP	1.21	2.56	1.88	0.64	3.30	4.12	3.63	2.27
GO_dr001P0015C16_F_ab1	AP2-EREBP	0.52	1.32	2.00	1.65	2.32	3.14	2.68	3.40
GO_drpoolB-CL3762Contig1 (*PhZFP1*)	Zinc finger protein (C2H2-type)	2.53	3.68	2.73	1.02	3.08	3.41	3.45	1.35
cn1485	Zinc finger protein (C2H2-type)	0.98	1.17	1.34	1.22	3.40	4.31	4.74	3.83
GO_dr004P0009O19_F_ab1	Zinc finger protein (RING-finger)	−0.31	−0.73	−1.87	−2.37	−0.55	−0.36	−0.62	−4.39
GO_drs31P0004P18_R_ab1	Zinc finger protein(C3H-type)	0.55	2.19	2.63	1.54	2.95	3.74	4.77	3.23
cn9788	RING-H2 subgroup RHE protein	1.29	2.07	1.62	0.62	2.98	4.09	3.64	0.88
cn6897	CCR4 associated factor 1-related protein	1.39	2.29	1.34	0.47	3.90	4.63	3.33	−0.11

*All data are shown log2 Ratio, and positive and negative values of Log2 Ratio are either up- or down-regulated genes in the four pair-comparisons*.

A total of 11 probes fell into three subfamilies of the zinc finger family (C2H2, C3H and B-box). Among these 11 up-regulated zinc finger proteins, we observed that the C2H2 subfamily was particularly enriched in cold-responsive transcripts. Additionally, another C2H2-type zinc finger TF (cn1485) was conspicuously up-regulated following 2 h, 24 h, and 5 d of cold treatment. Because the *P*-value was higher than 0.05, this TF was excluded from this analysis. However, its cold-induced expression, together with the other four zinc finger proteins (three C2H2 and one B-box), was verified by real-time RT-PCR (Table 3). We also identified one C3H-type zinc finger gene (GO_drs31P0004P18_R_ab1) whose expression level was evidently increased by cold stress and verified by real-time RT-PCR (Table 3).

The majority of MADS-box TFs detected by our array were up-regulated following 0.5 h or 2 h of cold treatment. Only FBP22 was found to be down-regulated after 2 h under cold stress. Interestingly, all of the MADS-box TFs were first identified to be involved in cold responses. Nine MYB or MYB-related family members showed differential expression, with five exhibiting cold-inducible and four cold-repressed expression. Several transcripts (i.e., cn2063 and cn8223) act as transcriptional regulators in phenylpropanoid metabolism. Five transcripts encoding the NAC domain transcription factor were identified, of which only one was down-regulated on the array. Similarly, four GRAS transcription factors detected on our array were up-regulated by cold, whereas one was down-regulated.

In addition to the above-mentioned TFs, we identified two HLHs (Helix-Loop-Helix) that were cold-repressed in our study. In contrast, two probes were found to encode SBP-box proteins, both of which were up-regulated. Additionally, three other types of transcription factor families, including AUX/IAA, HSF, and TCP, were also identified. Previous studies have linked some HSF TFs to cold stress (Carvallo et al., 2011).

### Genes responsive to cold and different abiotic stresses

To validate the results obtained through microarray analysis, real-time RT-PCR was conducted on 12 selected differentially expressed transcripts, most of which were transcription factors (e.g., AP2-EREBP, C2H2-type, and C3H-type zinc finger protein) and exhibited up-regulated expression (Table 2). The expression of 75% of the cold-responsive genes (9 of 12) based on the microarray data was confirmed using real-time RT-PCR. The log2 ratios from both the results of the microarray and the qRT-PCR analysis for these genes are shown in Table 3. Their expression kinetics from the real-time RT-PCR results were similar to those of the microarray analysis. These results re-confirmed the veracity of our microarray data. However, the fold changes of three cold-responsive genes measured by microarray and by real-time RT-PCR were not consistent. The relative low correspondence may be partially due to the low cut-off value used for the identification of DEGs.

The expression profiles of seven cold-responsive genes were analyzed by real-time RT-PCR using petunia *in vitro* shoot cultures treated with various stresses, including cold (2°C), dehydration, salinity (200 mM NaCl), osmotic (400 mM mannitol), ABA (100 μM), and JA (100 μM MeJA) for 3 h, 6 h and 12 h (Table 4). From these results, one gene, referred hereafter as *PhZFP1* (GO_drpoolB-CL3762Contig1), positively and exclusively responded to cold stress. This gene was predominantly up-regulated by cold and more or less down-regulated by other stresses. Several cold-responsive genes were also strongly induced by the dehydration, salt, osmotic, ABA, or JA stresses. For instance, the transcripts of two genes (GO_dr001P0015C16_F_ab1 and cn9788) were differentially accumulated under dehydration, salinity and osmotic stresses and were remarkably induced by ABA. Two genes encoding zinc finger proteins (cn1485 and GO_drs21P0009P06_F_ab1) were strongly induced by all of the abiotic stresses tested (including both ABA and JA) in this study.

**Table 4:** **Real-time RT-PCR verification of cold-responsive genes under various abiotic stresses**.

**Probe ID**	**Description**	**Cold**			**Dehydration**			**Salinity**			**Osmotic**			**ABA**			**JA**		
		**3 h/0 h**	**6 h/0 h**	**12 h/0 h**	**3 h/0 h**	**6 h/0 h**	**12 h/0 h**	**3 h/0 h**	**6 h/0 h**	**12 h/0 h**	**3 h/0 h**	**6 h/0 h**	**12 h/0 h**	**3 h/0 h**	**6 h/0 h**	**12 h/0 h**	**3 h/0 h**	**6 h/0 h**	**12 h/0 h**
GO_dr001P0015C16_F_ab1	AP2-EREBP	1.04	1.40	3.44	4.26	5.30	4.22	3.34	2.37	1.08	3.88	2.05	1.74	5.02	3.68	3.36	1.40	2.01	1.69
cn9788	RING-H2 subgroup RHE protein	3.45	3.95	4.29	1.67	2.66	2.03	1.94	2.12	2.04	2.21	3.38	3.19	5.20	4.04	3.27	0.64	1.59	2.11
cn6897	CCR4 associated factor 1-related protein	5.36	6.99	5.05	4.70	4.81	3.38	0.13	0.82	0.84	1.44	1.30	0.94	4.21	3.14	2.62	1.12	1.46	2.14
GO_drpoolB-CL3762Contig1 (*PhZFP1*)	Zinc finger protein (C2H2-type)	4.03	5.57	2.55	1.04	0.83	1.37	−0.99	−1.15	−2.44	−1.12	−0.30	−0.68	−1.12	−0.78	−0.93	−1.02	−0.72	−1.35
cn1485	Zinc finger protein (C2H2-type)	4.47	6.46	5.09	4.89	7.32	6.57	2.29	2.44	1.62	2.03	3.24	3.17	4.55	5.40	3.46	3.13	4.40	3.73
GO_drs21P0009P06_F_ab1	Zinc finger protein (B-box-type)	1.54	0.94	3.01	0.58	1.94	3.15	1.02	5.21	8.02	1.01	5.41	9.16	0.04	5.99	7.91	1.02	6.12	8.70
GO_drs31P0004P18_R_ab1	Zinc finger protein (C3H-type)	2.94	3.95	3.10	1.49	2.32	2.71	0.71	0.84	2.05	1.84	1.48	1.92	0.96	1.44	0.54	0.94	0.76	1.80

*Seven selected cold responsive genes were analyzed under different treatments, including cold (2 °C), dehydration, salinity (200 mM NaCl), osmotic (400 mM mannitol), ABA (100 μM), and JA (100 μM MeJA) for 3, 6, and 12 h, respectively. All data are shown log2 Ratio, and positive and negative values of Log2 Ratio are either up- or down-regulated genes in the three pair-comparisons*.

### Network classification and identification

**Figure 2:**
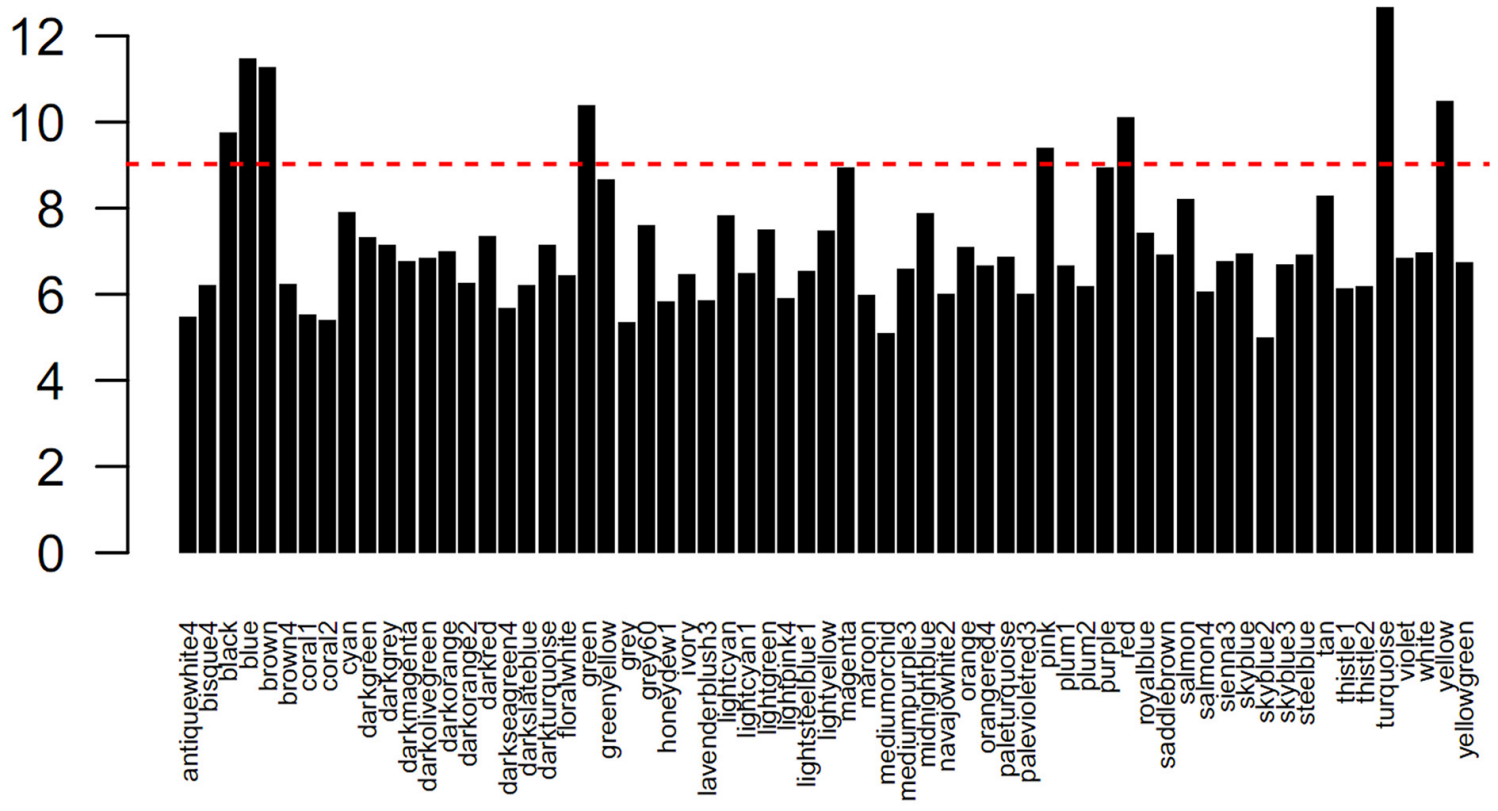
**Distribution of genes (probes) in each module**. The red line indicates the number of 500 genes.

**Figure 3:**
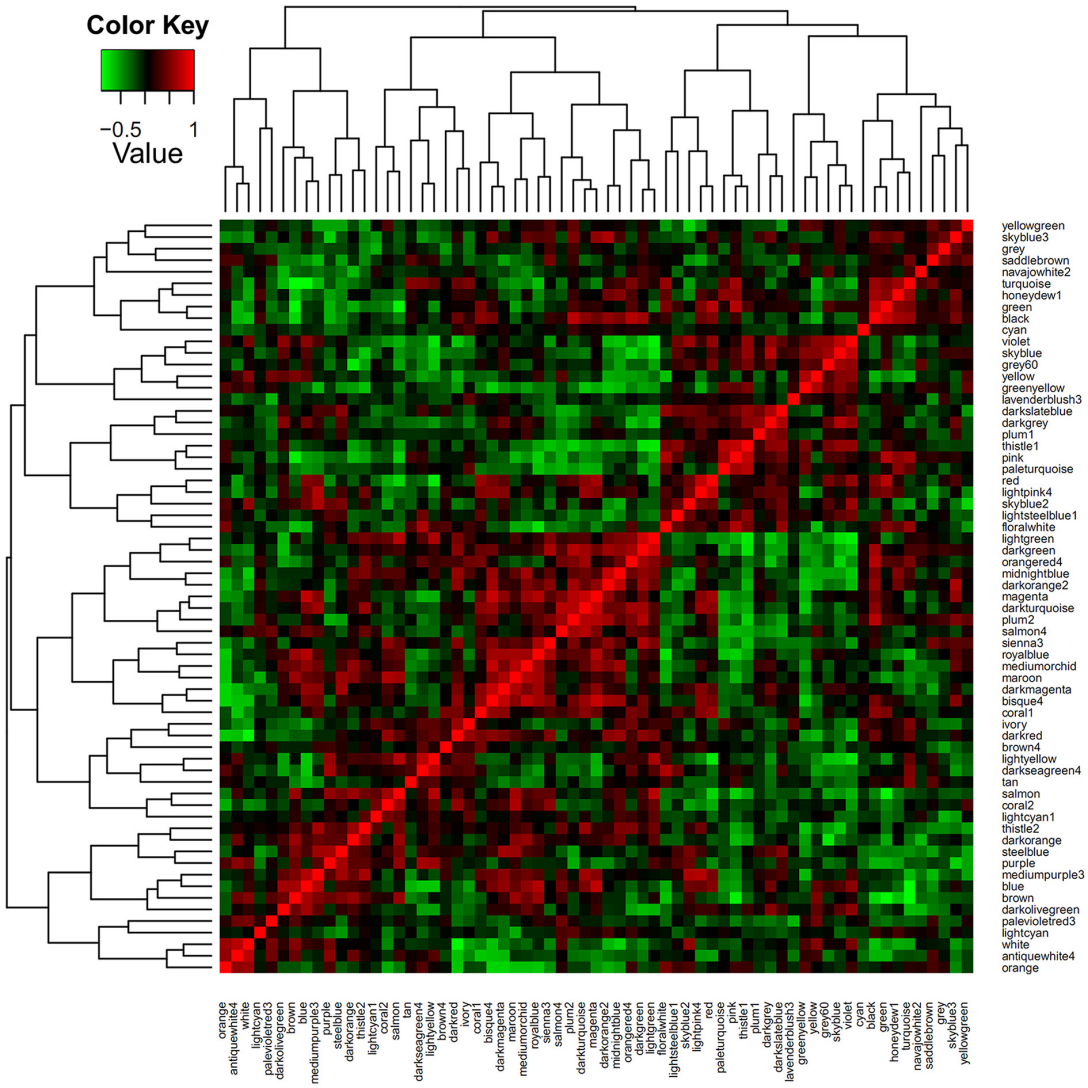
**Module eigenvector clustering**. The co-expression network with 65 modules and the eigenvectors of each module calculated and clustered using the WGCNA software. The color scale represents the strength of the correlation between the modules (from −1 to 1).

To identify sets of functionally related genes, we constructed a petunia gene co-expression network using our microarray samples as input. Prior to the analysis, the gene numbers of each module were transformed to the log2 base scale. Then, a clustering approach of the weighted correlation network was undertaken, resulting in a total of 65 modules of highly correlated genes. In Figure 2, the modules are denoted by different colors. Obviously, the numbers of genes (probe sets) per module varied, and more than half of the modules contained less than 500 genes (probe sets) (Table S4). To explore the co-expression relationships between modules, a module's representative expression pattern was summarized using the first principal component of all of the module's gene members. Using the complete linkage method, all of the module eigengenes were clustered, thereby allowing the characterization of similar structures between the modules (Figure 3).

Among the 65 modules, the purple module (size 491) strongly attracted our attention. This module included several transcription factors such as PhZFP1 (GO_drpoolB-CL3762Contig1) and other zinc finger TFs (cn1485 and GO_drs31P0004P18_R_ab1) displaying substantial responsiveness to cold stress in our analysis. *PhZFP1* was regarded as a particularly promising candidate gene worthy of further investigation based on its unique stress-induced expression profile compared with other cold-responsive genes tested in our study (Table 4). To quantify the association between *PhZFP1* and other petunia genes in the purple module, the top 50 genes positively correlated with *PhZFP1* were chosen and are shown in Table S5. Strikingly, *PhCBF1* (GO_dr004P0029M08_F_ab1) was also found to be contained in the purple module and included in the top 50 genes positively correlated with *PhZFP1*. Because *PhZFP1* and *PhCBF1* were included in the same module and positively correlated with each other, they most likely have a certain relatedness in functionality.

## Discussion

### Relationship between flavonoid metabolism and cold stress response in petunias

The results of the biological process for functional enrichment analysis showed that the categories associated with flavonoid metabolism, including “pigment biosynthetic process” and “flavonol metabolic process,” were the most remarkable GO terms. This result suggests that the activation of flavonoid metabolism at the relatively late stage may be an integral part of the plant adaptation to low temperatures in petunias. Previous work has mainly indicated that flavonoids are induced in response to UV irradiation. It was considered that these UV-absorbing compounds could provide protection against cell death stemming from UV-B damage by protecting DNA from dimerization and breakage (Dixon and Paiva, 1995). Other stresses such as cold that induce phenylpropanoids have been less well studied. Increased anthocyanin levels were detected following cold stress in blood oranges (Crifo et al., 2011), but the reasons for this increase are still unclear. In this study, several genes encoding key enzymes implicated in flavonoid metabolism were found among the cold-induced genes at 5 d/0 h (Table S2), such as CHS (chalcone synthase, GI_TC432), CHI (chalcone isomerase, cn302) and F3H (flavanone 3-hydroxylase, cn2956) (Table S2). These genes are held in common in the biosynthetic pathway of anthocyanins, flavonols, and flavan-3-ols. Our results in petunias are to a great extent consistent with previous observations concerning the increased accumulation of two flavonoid pathway proteins (CHS and F3H) in strawberries in response to cold stress (Koehler et al., 2012). Thus, we speculate that the well-defined induction of these genes in petunias subjected to cold stress would probably lead to a rise in flavonoid levels; in turn, the rising flavonoid levels might contribute to the control of cell osmotic potential, thereby effectively coping with the imposed adverse conditions (Chalker-Scott, 1999). However, it is worthwhile to note that in the present study the noticeable major category predicted to be involved in the cold stress response in petunias was associated with flavonoid metabolism, whereas other significant cold-related GO terms were rare. This may be because a large portion of DEGs share homology with genes encoding putative proteins of unknown function or share no significant homology with any database accession. Future studies will be focused on detailing the quantitative and qualitative transcriptomes of petunias under cold stress. These efforts will be greatly facilitated by the completion of petunia genome sequencing and the rapidly developing technologies for RNA sequencing.

### Established and novel players involved in cold stress regulation in petunias

Transcription factors play important roles in plant development and stress tolerance (Lee et al., 2005), and genes related to “Transcription factor activity” may be central regulators involved in upstream cold signal transduction which are capable of triggering a cascade of downstream gene expression. In this study, we found 61 transcription factor genes that were differentially regulated across different time points. The six most highly represented TF families were AP2-EREBP, zinc finger, MADS-box, MYB/MYB-related, NAC, and GRAS. These cold-responsive TFs (shown in Table 2) represent potentially important regulators of the cold-stress response in petunias.

The AP2-EREBP family plays a predominant role in the early stages of the cold response, which has been evidenced by some of the best characterized CBFs (CRT/DRE binding factors) in the cold regulatory pathway. CBF transcription factors, attached to the A-1 subfamily of the ERF/AP2 TF family, are major regulators that exert their effects via activation of cold-regulated effectors in *Arabidopsis* and other plants (Gilmour et al., 1998). Here, one *CBF* homologous gene (GO_dr004P0029M08_F_ab1) *PhCBF1*, was highly induced, suggesting that the response of the DREB/CBF pathway to cold in petunias is at least partially similar to that observed in other plants, although it is probably not the only pathway involved. Certain member of the AP2-EREBP family has been reported to show responses to cold stress in *Capsicum annuum*, such as CaEREBP-C2 (http://www.ncbi.nlm.nih.gov/protein/AAX20035). Other members were first identified as being involved in the cold response, such as NtERF5, which was previously characterized as a pathogen-regulated factor (Fischer and Droge-Laser, 2004). Additionally, *NsERF3*, which is homologous to two AP2/EREBP family members (cn9604 and GO_dr001P0015C16_F_ab1), was shown to be involved in the regulation of gene expression by stress factors as well as components of stress signal transduction pathways. This gene most likely acts as a transcriptional repressor and may regulate other AtERFs (based on similarity), whereas the regulation mechanism remains obscure (Kitajima et al., 2000), it might be of interest to further study these two AP2/EREBP TFs.

Zinc finger family members in *Arabidopsis* have been demonstrated to be involved in the cold response (Lee et al., 2005). Recently, the genes encoding zinc finger proteins in blueberries were also detected to be cold acclimation-responsive based on transcriptome database mining (Die and Rowland, 2014). In our study, we identified 11 up-regulated zinc finger proteins, among which members of the C2H2 subfamily were especially abundant. Previous studies revealed that many C2H2 zinc finger proteins regulate responses to multiple abiotic stresses, such as ZAT10 and ZAT12 in *Arabidopsis* (Davletova et al., 2005; Mittler et al., 2006). Thus, C2H2 zinc finger proteins are crucial factors that link various signal transduction pathways in response to different stresses and participate in several cellular processes, whereas only a few C2H2 proteins seem to be associated with responses to one specific stress (Kielbowicz-Matuk, 2012). Consistent with this finding, several petunia cold-responsive C2H2-type zinc finger genes found in our array were induced not only by cold stress but also by other abiotic stresses, including dehydration and salinity. The exception was one C2H2-type zinc finger gene called *PhZFP1* (GO_drpoolB-CL3762Contig1). *PhZFP1* was exclusively and positively responsive to cold. Interestingly, our findings that *PhZFP1* and *PhCBF1* were contained in the same module and tightly correlated with each other suggested that this gene may be a critical regulator of the cold stress response that functions in an ABA-independent manner similar to *CBFs*. Abundant evidence has illustrated that the transcription network of CBF plays a significant role in cold acclimation of evolutionarily different plant species. Nevertheless, gene expression analysis revealed several transcription factors besides CBFs that are induced during cold acclimation. Transgenic analysis of cold-inducible TFs helped to validate their functions in cold tolerance and suggested that other classes of transcription factors also play a significant role in cold acclimation (Chinnusamy et al., 2010). For example, constitutive overexpression of the soybean C2H2-type zinc finger *SCOF1* (soybean cold-inducible factor-1) in *Arabidopsis* transgenic plants elevated the expression of *COR* (cold-regulated) genes and conferred constitutive freezing tolerance. SCOF1 interacts with soybean G-box binding bZIP transcription factor SGBF1, which is induced by both cold and ABA. It was suggested that SCOF-1 might function as a positive regulator of *COR* gene expression mediated by the ABA responsive element via protein-protein interactions, which in turn improves cold tolerance of plants (Kim et al., 2001). The large proportion of C2H2-type zinc finger family members with cold-inducible transcripts identified in our study makes this class of TFs an attractive target for further functional characterization. For instance, the question of whether *PhZFP1* is involved in the CBF-dependent or CBF-independent transcriptional pathway and the mechanism by which the *PhZFP1* transcriptional networks operate during cold acclimation and cold stress tolerance in petunias are unknown. Compared with the relatively large C2H2 subfamily, knowledge about the biological role of the smaller C3H and B-box subfamilies is very limited. C3H-type zinc fingers are a type of plant cold shock domain protein (CSDP). Although their biological function and modes of action are not totally revealed, evidence from *Arabidopsis* suggests that CSDPs may confer cold tolerance by functioning as RNA chaperones (Kim et al., 2007, 2009). All of these results suggest an important role for zinc finger genes in petunia responses to cold stress.

The notable MADS-box family members are known to be key regulators of several plant development processes. The best studied plant MADS-box TFs are those involved in floral organ identity determination (Parenicova et al., 2003). However, a recent study of the *Arabidopsis* MADS-box gene SUPPRESSOR OF OVEREXPRESSION OF CONSTANS1 (*SOC1*) revealed that this representative floral activator could also function as a negative regulator of the cold response pathway through the direct repression of *CBFs* (Seo et al., 2009). This result hints that the responsiveness of the MADS-box transcription factors to cold stress observed in this study might not be a coincidence, and offers the possibility that some MADS-box TFs, for instance, FBP22 (GI_NP1240016) which displays high homology to SOC1 and other SOC1-like proteins are the key regulators of crosstalk between the cold response and flowering time regulation. MYB proteins are crucial factors controlling development, metabolism and responses to biotic and abiotic stresses in regulatory networks (Dubos et al., 2010). A comprehensive analysis of MYB transcription factor gene expression demonstrated that almost all MYB TFs are responsive to hormones or stresses (Chen et al., 2006). In this study, five up-regulated and four down-regulated MYB or MYB-related family members showed diverse expression patterns. One MYB-related transcription factor (GO_dr004P0017J05_F_ab1) was reported to be a regulator of the tobacco defense-related genes (Sugimoto et al., 2000). Several transcripts, such as cn2063 and cn8223, were identified to be involved in the regulation of anthocyanin biosynthesis (Nakatsuka et al., 2008) (http://www.ncbi.nlm.nih.gov/protein/ABY40371). The differential expression of MYB TFs implies that overlapping environmental pressures or certain metabolic pathways may be integrated into petunia responses to cold stress mediated by the regulation of MYBs.

The appearance of five transcripts encoding the NAC domain TF on the list of transcription factors regulated in response to cold stress is in line with the existing knowledge concerning plant-specific NAC proteins, which are renowned for their roles in biotic and abiotic stress responses (Puranik et al., 2012). In contrast, the currently available knowledge concerning the function of the GRAS gene family is not abundant. Most characterized members of this family encode transcriptional regulators which have functions in plant growth and development, such as axillary meristem formation, root radial patterning and gibberellin signal transduction (Tian et al., 2004). It is worth noting that there have been some results supporting a function for GRAS TFs in plant responses to biotic and abiotic stresses (Kim et al., 2001).

An additional five families (HLH, SBP-box, AUX/IAA, HSF, and TCP) accounted for approximately 11.5% of the total number of cold-responsive TFs detected in our array. Although these families play various roles in plant developmental processes and environmental responses, for instance, the SBP-box gene family has been revealed to control flower and fruit development as well as other significant physiological processes (Chen et al., 2010), most have not been linked to cold stress resistance in plants. Nevertheless, it is important to note the knowledge concerning the function of some members in other aspects has started to gradually increase. For example, several grape SBP-box genes were identified and presumed to be potentially involved in the defense against different stresses, including cold treatment (Hou et al., 2013).

A great number of genes (approximately 37.8% of the total 2071 DEGs) encode proteins of unknown function. The study of these genes may reveal new mechanisms that are fundamental to petunia plants coping with low temperatures. Indeed, the microarray that was used to monitor gene expression was based on ESTs from *Petunia hybrida* and *Petunia axillaries*, which was not sufficiently reflective of the overall genome. Thus, the present analysis was significantly limited in scope. Nevertheless, our results support the fact that the large-scale dataset strategy can be useful in obtaining meaningful biological information. In this sense, it is possible that our results have identified for the first time a set of potential regulators which may play a role in the regulation of cold stress tolerance in petunias. Further investigations will be focused on the functional validation of candidate genes for improving petunia tolerance through genetic engineering.

### Crosstalk among different signaling systems in regulating the cold stress response in petunias

In plants, the existence of complex signaling networks and multiple defense strategies results in their enhanced defense capacity. Because the cold stress-signaling pathway may interact with other signaling systems (i.e., drought, salt, and ABA) (Seki et al., 2002), we conducted qRT-PCR to investigate the expression patterns of seven cold-responsive genes under different abiotic stresses. Most were induced to varying degrees by at least one of the five common abiotic stress treatments, including exposure to dehydration, salinity, osmotic, ABA, and JA in addition to cold, suggesting that they may participate in the regulation of a wide spectrum of responses to diverse abiotic stresses. These results indicate that dynamic crosstalk exists among signaling pathways associated with cold, dehydration, and salt stress in petunias, which may induce common alterations, such as cellular dehydration (Rabbani et al., 2003).

Induced defense responses are controlled by a network of interconnected signal transduction pathways in which hormonal signals, such as ABA or JA, could coordinately activate the transcription of various defense-related genes (Glazebrook, 2005). Plant hormones offer a vital function in signaling networks implicated in plant responses to a variety of biotic and abiotic stresses. ABA plays a critical role in the abiotic stress response by inducing stomatal closure which results in the reduction of transpiration (Pantin et al., 2013) and by regulating root growth, ion channels and gene expression (Duan et al., 2013), whereas JA plays an important role in the defense response, especially against biotic stresses, such as necrotrophic pathogens and wounding (Thaler et al., 2012). In this study, we determined that the expressions of the majority of the cold-responsive genes analyzed (5/7) were significantly modified by ABA treatment (Table 4). Thus, our result is in agreement with the known effect of ABA on cold stress signaling, which indicates their possible involvement in ABA-dependent pathways. However, recent findings have increasingly suggested that JA also plays a role in the regulation of plant abiotic stress tolerance, including stresses, such as salinity and cold (Kang et al., 2005; Khan et al., 2012). These findings may explain our observation of the substantial elevated expression level of two cold-responsive zinc finger genes (cn1485 and GO_drs21P0009P06_F_ab1) following MeJA treatment (Table 4). For example, it was demonstrated that jasmonate acts as an upstream signal of the ICE-CBF/DREB1 transcriptional pathway to positively regulate freezing stress responses in *Arabidopsis* (Hu et al., 2013). Because the two zinc finger protein genes mentioned above were also responsive to all of the other abiotic treatments, the result suggests that their involvement in the response to cold stress may be dependent on multiple signaling pathways including MeJA molecular signals. Taken together, our analyses indicate that crosstalk among different signaling systems plays a significant role in regulating the cold stress response in petunias.

### Conflict of interest statement

The authors declare that the research was conducted in the absence of any commercial or financial relationships that could be construed as a potential conflict of interest.

## Supplementary material

Table S1 **Primers used for real-time RT-PCR verification**.

Table S2 **Differentially expressed genes (DEGs) in the four paired comparisons from low temperature-treated samples at 0 h, 0.5 h, 24 h and 5 d**.

Table S3 **Gene ontology (GO) terms enriched by cold responsive genes at all time points**.

Table S4 **Gene modules detected by WGCNA in the petunia gene co-expression network**.

Table S5 **The top 50 genes positively correlated with *PhZFP1***.
